# The water footprint of irrigation-supplemented cotton and mung-bean crops in Northern Ethiopia

**DOI:** 10.1016/j.heliyon.2021.e06822

**Published:** 2021-04-22

**Authors:** Filmon Tquabo Gebremariam, Solomon Habtu, Eyasu Yazew, Berhane Teklu

**Affiliations:** a Department of Land Resources Management and Environmental Protection, Mekelle University, Tigray, Ethiopia; b Institute of Water and Environment, Mekelle University, Tigray, Ethiopia; c Hiwot Agricultural Mechanization P.L.C, Humera, Tigray, Ethiopia

**Keywords:** Water footprint, Center-pivot systems, Cotton, Mung-bean, CROPWAT, Ethiopia

## Abstract

Global freshwater resources are getting scarcer and scarcer due to the ever-increasing population, climate change, and other human activities. Hence, assessing the consumption of freshwater by different consumers is a key to efficiently utilize the resource. In this study, the Water Footprint Assessment (WFA) tool was used to determine the water footprint (WF) of Center Pivot (CP) irrigated cotton and mung-bean production using two approaches, namely, CROPWAT and field-data based methods. Based on the CROPWAT-based estimates, the average total WF of cotton was found to be 2745 m^3^/ton. Out of this, the green and blue WF contributed to an average of 35% and 65 %, respectively. For mung-bean, the total WF was 6561m^3^/ton, of which blue WF covered around 93 %. Comparison of the blue WF from CROPWAT and field-data based estimates showed a good agreement (nRMSE = 4.5 %, nMBE = 10.7 % and relative error/RE/ranging from 0.8 to 17% for cotton and 12.6% for mung-bean) and no significant difference (p = 0.456) was obtained between the two estimates. The effect of planting date on the WF estimation also showed a small variation of 0.7%–6.6 % for cotton and up to 12% for mung-bean. However, major reductions were obtained on the blue WF of cotton and mung-bean as a result of changing planting dates by about two months prior to the baseline planting dates. In this study, it is concluded that WF assessment could be satisfactorily estimated using CROPWAT model if supported with field obtained information such as soil, crop, and weather data. Another finding of the present study was that, changing planting dates close to the major rainy months could substantially contribute to reducing the blue WF in similar climates.

## 1 Introduction

Globally, the water use rate over the last century has grown by more than twice the population increase. Agriculture, with an average freshwater withdrawal of 70%, is the largest consumer of global freshwater resources (FAO, 2012). The freshwater consumption of agriculture in the African continent is estimated at 84.1 % (FAO, nd). Over the past 30 years, population growth has led to an increase in food consumption by 100%. FAO projections indicate that the food demand will increase to about 60% by 2050 (Alexandratos and Bruinsma, 2012). Besides, agriculture is the major contributor to water pollution due to the application of fertilizers, pesticides, and other chemicals. Hence, the scarce freshwater resources are getting scarcer by the ever-increasing population, climate change, and other human activities.

The arid and semi-arid regions of Nothern Ethiopia are one of the water-scarce regions of Eastern Africa. To avert water scarcity, the Ethiopian government had embarked massive investments in the development of water resource projects to put more land into agriculture (Hagos et al., 2012). However, the sustainability of the limited water resources, among many other factors, is being threatened by poor water management practices (Kifle and Gebretsadikan, 2016). Various on-farm water management studies have been conducted to improve the water management practices in irrigated agriculture in the region. Nonetheless, the majority of the studies focus on the management of blue water alone, ignoring the importance of the green water resources (Schuol et al., 2008). The WFA tool is very powerful in identifying the proportion of freshwater consumption to improve water security and water use efficiency in irrigated agriculture.

The WFA is a general tool, introduced by Hoekstra and Hung (2002) for assessing the consumption of freshwater by different products along their supply chain. Nowadays, the WFA tool is gaining an increased applicability in determining the consumption of freshwater by crops (rain-fed or irrigated). For crop production, WF is the amount of freshwater used by a crop during the whole growing period (Xinchun et al., 2018). The three components of WF in crop production are; the green, blue, and grey WF. The green and blue footprints refer to consumptive use (evapotranspiration) of water by plants, whereas the grey WF refers to the amount of water used to assimilate the pollutants as a result of fertilization or the existing water quality standards. The WF approach gives more focus on the consumptive use (evaporative demand) of water by plants rather than the amount of water withdrawn from the source (Hoekstra, 2003).

Due to its increased merits, the WF approach has attracted the attention of many researchers over the past few years for a wide variety of applications (Xinchun et al., 2018). Among which, the application of WFA tool for assessing freshwater use by crops has become very popular (Chukalla et al., 2015; Geng et al., 2019; Herath et al., 2014; Madugundu et al., 2018; Mekonnen and Hoekstra, 2011; Morillo et al., 2015; Rodriguez et al., 2015). Many of these studies on WF of crop production were focused on either global (Aldaya et al., 2010; Chapagain et al., 2006) or national scales (Ahmed and Ribbe, 2011; Tsakmakis et al., 2018). Moreover, the majority of these studies employ conceptual/mathematical models to simulate the soil-plant-water interactions and assess the WF components. Although models save time and energy, the use of field data and national statistics is likely to improve WF assessment of crop production. When compared to model-based estimates, WF assessments based on field measured data are believed to give more realistic results (Karandish and Šimůnek, 2019). So far, WF assessments of crop production based on field measurements are very few (Castellanos et al., 2016; Herath et al., 2014; Xinchun et al., 2018). Besides, the process of calibrating and comparing model-based assessment of WF against field measurements remains immature (Hoekstra, 2017). Furthermore, most of such models basically employ climate, soil and crop datasets, which vary depending on the geographical locations (Aldaya et al., 2010; Lovarelli et al., 2016; Zhuo et al., 2014), justifying the local assessment of WF for better results., On the other hand, WF assessments made at regional, national or global scale mostly use input data such as precipitation and temperature obtained from remote sensors which often result in course resolution WF estimates.

Many studies have employed the WFA tool to investigate the effects of different management interventions on water consumption of crops. Some of these studies involve improvements in water-saving irrigation technologies, optimization, and adjustments on planting strategies believed to reduce the WF from the agricultural sector (Jin et al., 2016). For example, Chukalla et al. (2015) and, Karandish and Šimůnek (2019) compared the effect of different combinations of mulching, irrigation strategies and methods on the water footprint of winter wheat (Jin et al., 2016); Wang et al. (2015) in thier model simulations obtained a 3.4% reduction in groundwater-based blue WF by shifting the sowing area from north to the south part of the North China Plains (NCP). Zhuo et al. (2014) also found diminished total and blue WF as a result of late planting dates of four crops (i.e. maize, rice, wheat, and soybean). However, the impact of different water-saving and management strategies on the total and components of WF in sub-Saharan Africa remains scarce.

This study was conducted in an attempt to address the water management issues in irrigated cotton and mung-bean production, and reducing the uncertainties in WF assessment of crop production. Hence, the major objectives of this research were to: (1) quantify the WF of cotton and mung-bean production with regard to local climates. (2) compare the blue WF assessment based on CROPWAT model and those obtained from field data and, (3) model the impact of planting date scenarios on the WF of the two crops in a recently established center-pivot irrigated system in Northern Ethiopia.

## 2 Materials and methods

### 2.1 Description of the study area

**Figure 1:**
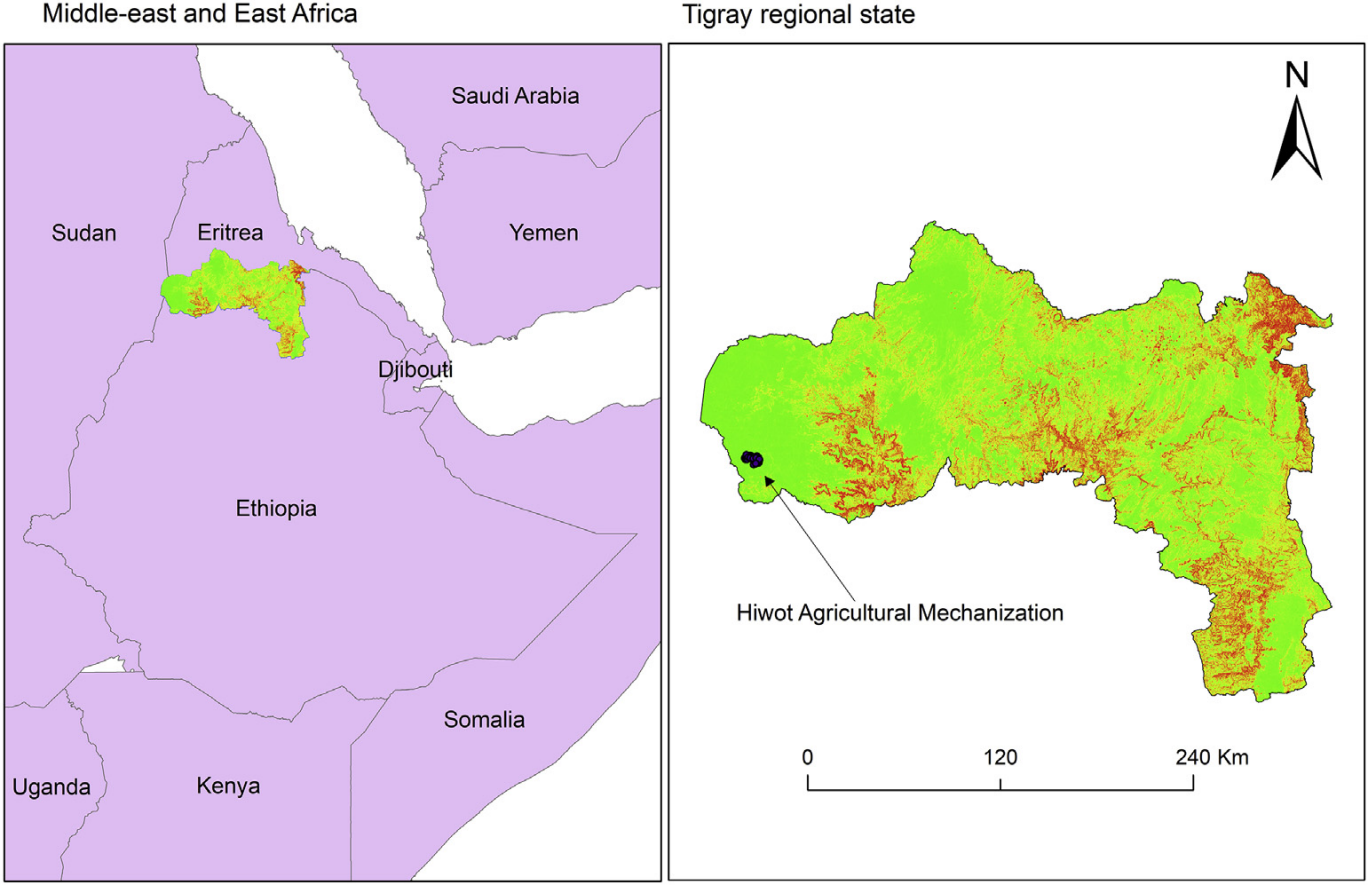
Location of HAM.

**Figure 2:**
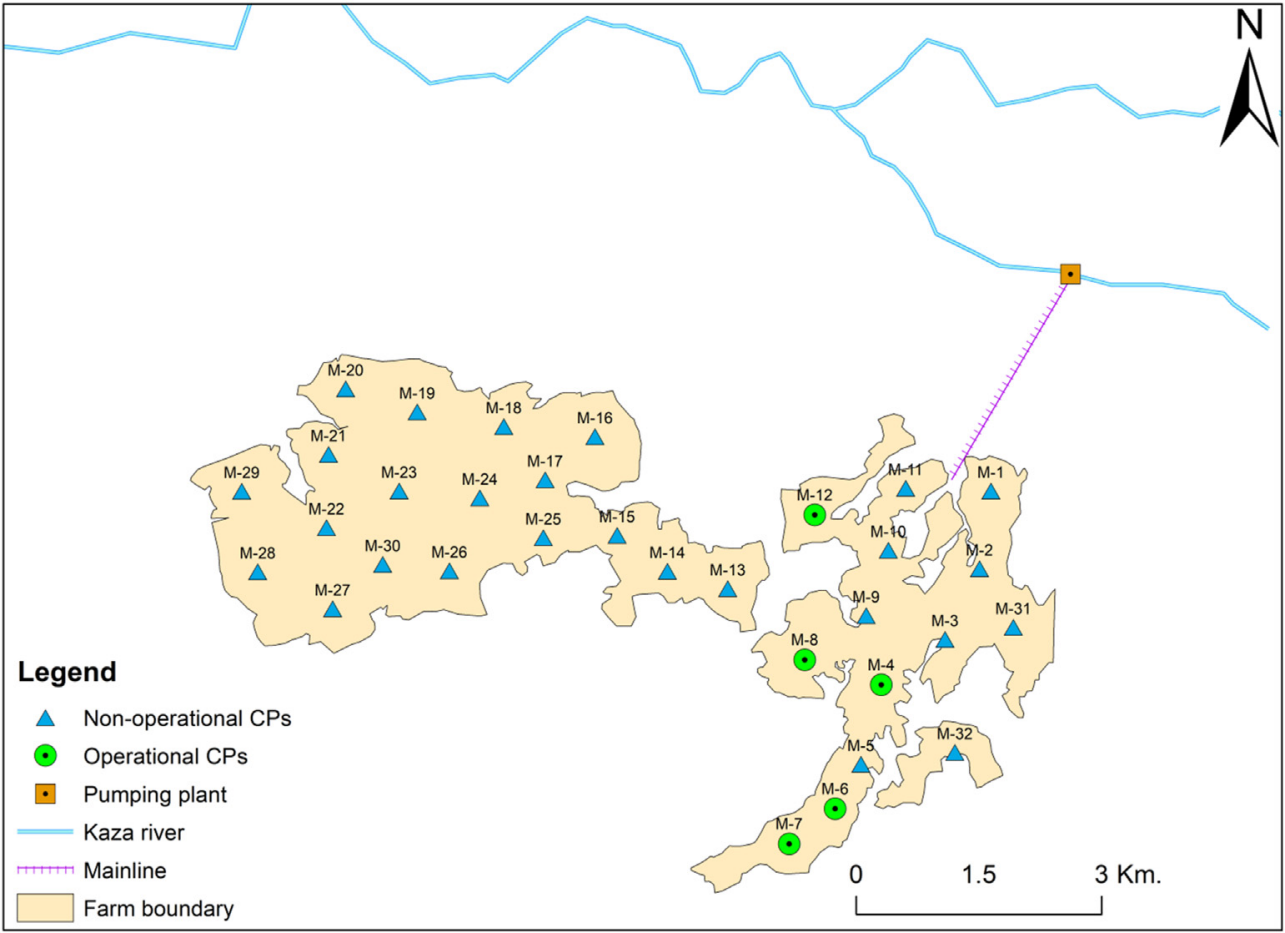
Layout of the areas irrigated by Center-pivot machines (operational CPs are those CPs which were active during the study year).

The study site, Kebabo farm, is owned by Hiwot Agricultural Mechanization P.L.C (HAM). The farm is situated in Tsegede district, Western zone of Tigray, Northern Ethiopia. Geographically, it is found at 36° 40′ 38″ E and 12° 20′ 43″ N (Figure 1). The altitude of the area ranges from 550 to 650 m above sea level. The mean annual rainfall in the area is 635 mm. The area is known for its hot climate with a mean daily temperature ranging from 20-35 °C. The predominant soil textures in the study site are clay and clay loam. The source of irrigation water for Kebabo farm is diverted from Kaza River. Kaza river is an ephemeral river with a mean annual flow of 490 Mm^3^/year. The pumping station is located at a distance of 3 km from the nearest CP field (Figure 2).

The land use of the Kaza river watershed is 70% cultivated land, 10% forest and woodland, 3% covered with forest land, 3% shrub grassland, 4 % grassland and the rest 10% covered by bare/rocky surface (HAM, 2009).

The gross cultivated area in Kebabo is around 2400 ha. Out of which, 1730 ha is designed to be irrigated by a newly introduced CP irrigation system. The major crops cultivated in the area are cotton, sesame, sorghum and recently mung-bean. Globally, Ethiopia is the 13^th^ largest producer of cotton. The annual cotton production of Ethiopia in the 2017/18 season was 53000 tons (Global Agricultural Information Network, 2019). Tigray Regional State is one of the cotton production regions in northern Ethiopia with an estimated potential cultivable area of 269,130ha and an average yield of 1.5 ton/ha (Gudeta and Gebreegziabher, 2019). According to the Central Statistical Agency of Ethiopia (2018), the total area covered by mung-bean in Ethiopia during the 2017/2018 production season was more than 40,000 ha and an average yield of 0.5 ton/ha. With around 60 % of global production, India is the largest producer of mung-bean, followed by China and Thailand (International Market Analysis Research and Consulting, 2019).

### 2.2 Description of Center Pivot (CP) machines

Kebabo farm was established with a vision to develop the area with the help of mechanized agricultural systems. For this purpose, the company introduced a modern CP irrigation system. Hence, around 1730 ha area of the farm was designed to be irrigated by using thirty-two (32) CP machines each capable of irrigating an area ranging from 20-70 ha. However, due to various reasons such as breakage, and water supply shortages, only five CP machines (M-4, M-6, M-7, M-8, and M-12) were functional during the study period (2017/18). All the CP machined were not equipped with end gun. The layout of the CP machines is depicted in Figure 2. The CP machines considered in this study are indicated by green circles. The detailed description of each CP machine including their irrigable area, average application rates, and operating pressures are presented in Table 1.

**Table 1:** Description of the five CP machines (source: Omni, Inc.).

CP Machine code	Irrigable Area (Ha)	Total delivered flow (lps)	Inlet pressure (bar)	End pressure (bar)	Average application rate (mm/hr)	No. of spans	Total length (m)	Motor loaded speed (RPM)
M-4	50	60.79	2.00	1.00	0.43	7	401.42	1425
M-6	30	36.38	1.23	1.01	0.43	5	309.00	1425
M-7	40	48.55	1.25	1.01	0.43	6	356.92	1425
M-8	50	60.79	2.00	1.00	0.43	7	401.42	1425
M-12	60	73.01	2.55	1.03	0.43	8	437.39	1425

NB: Ha-hectares, lps - liters per second, RPM-revolutions per minute.

### 2.3 Data inventory

Data regarding daily application depths, duration, and frequency of application were recorded during the 2017/18 season. Table 2 presents the monthly depth of water applied for each CP machines (one for mung-bean and five CP for cotton) obtained during the 2017/2018 season. The 50 ha area irrigated by CP machine M-4 was covered with 45 ha cotton and 5 ha mung-bean. These crop coverages will be assigned, hereafter, as M-4C and M-4M to represent the cotton and mung-bean planted in CP machine M-4. At a normal irrigation event, the CP machines were calibrated to apply 10.5 mm of water for a period of 23.3 hrs. However, sometimes the application depth may be needed to stay for a shorter period of time. In such cases, application depths were obtained by interpolation based on a linear relationship between the depth of water (mm) applied and duration (hrs) as recommended by the manufacturer.

**Table 2:** Monthly schedule and depth of water applied during 2017/18 season for each crop and CP machine.

Month	Depth of water applied (mm)					
	M-4C (cotton)	M-4M (mung-bean)	M-6 (cotton)	M-7 (cotton)	M-8 (cotton)	M-12 (cotton)
Sep	27	27	27	27	0	0
Oct	68	68	68	68	96	96
Nov	150	150	137	150	137	150
Dec	82	82	68	55	68	41
Jan	88	88	88	69	47	0
**Total**	**416**	**416**	**389**	**369**	**347**	**287**

### 2.4 CROPWAT input data

CROPWAT model requires three major inputs which could be categorized under climatic, crop and soil datasets.

#### 2.4.1 Climatic data

**Figure 3:**
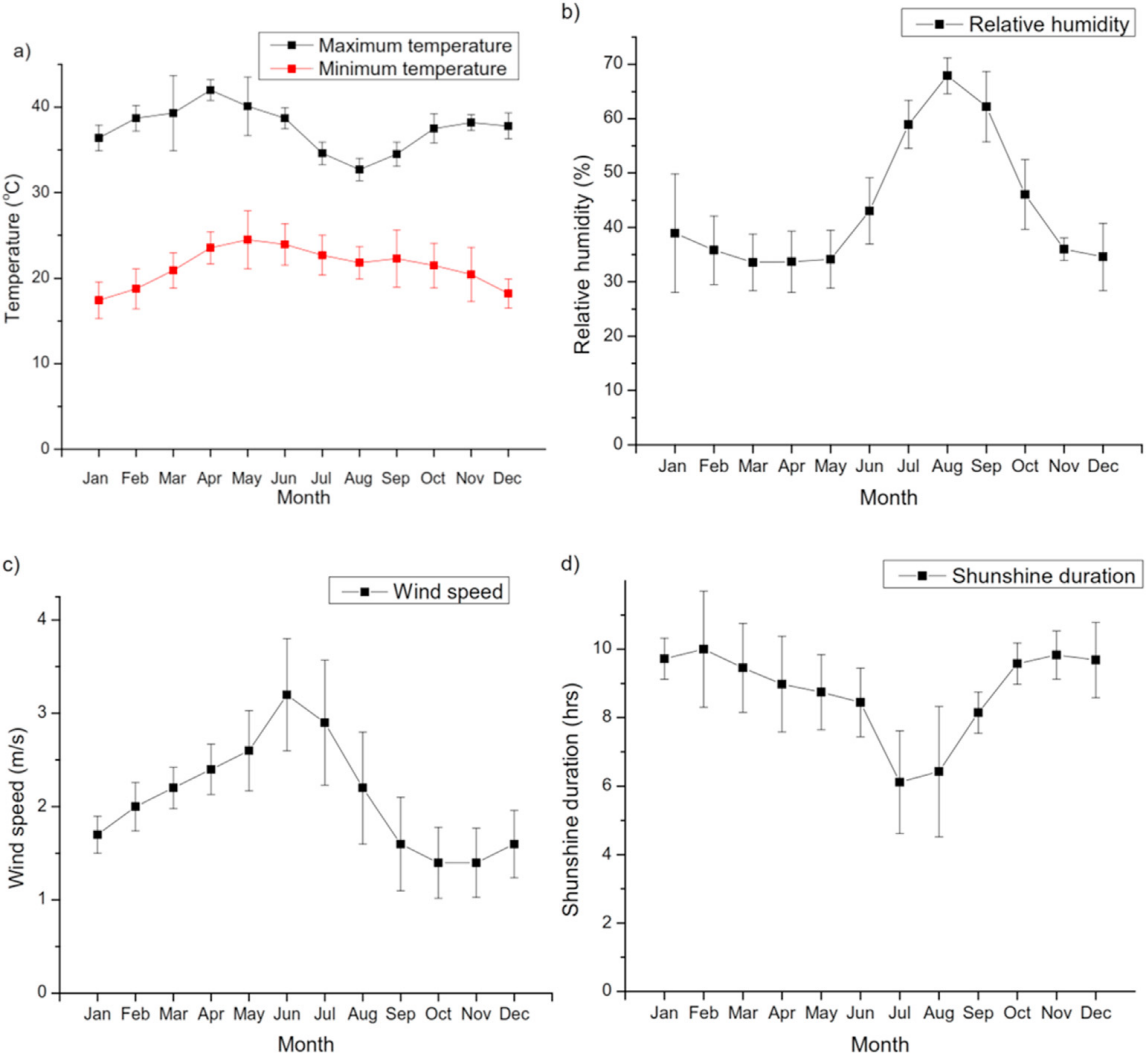
Mean monthly values of minimum and maximum temperature (a), relative humidity (b), wind speed (c) and sunshine duration (d) in the study site.

The main climatic datasets required by CROPWAT are average monthly values of rainfall, minimum and maximum temperatures, wind speed, relative humidity, and sunshine hours. Climatic data were obtained from Ethiopian Metrological Agency (EMA) and HAM. The trends of average monthly minimum and maximum temperature, relative humidity, wind speed, and duration of sunshine hours are presented in Figure 3. The mean daily maximum air temperature ranged from 32.7 °C (August) to 42 °C (April), whereas the mean daily minimum temperature ranged from 17.4 °C in January to 24 °C in May with a mean temperature of 21 °C. The average relative humidity was 43.7 %, with the peak values occurring from August to September and minimum values from March to April. The wind speed in the area is was lowest in November and highest in June. The duration of sunshine hours ranged from 6.1 to 10, with an average value of 8.7.

**Figure 4:**
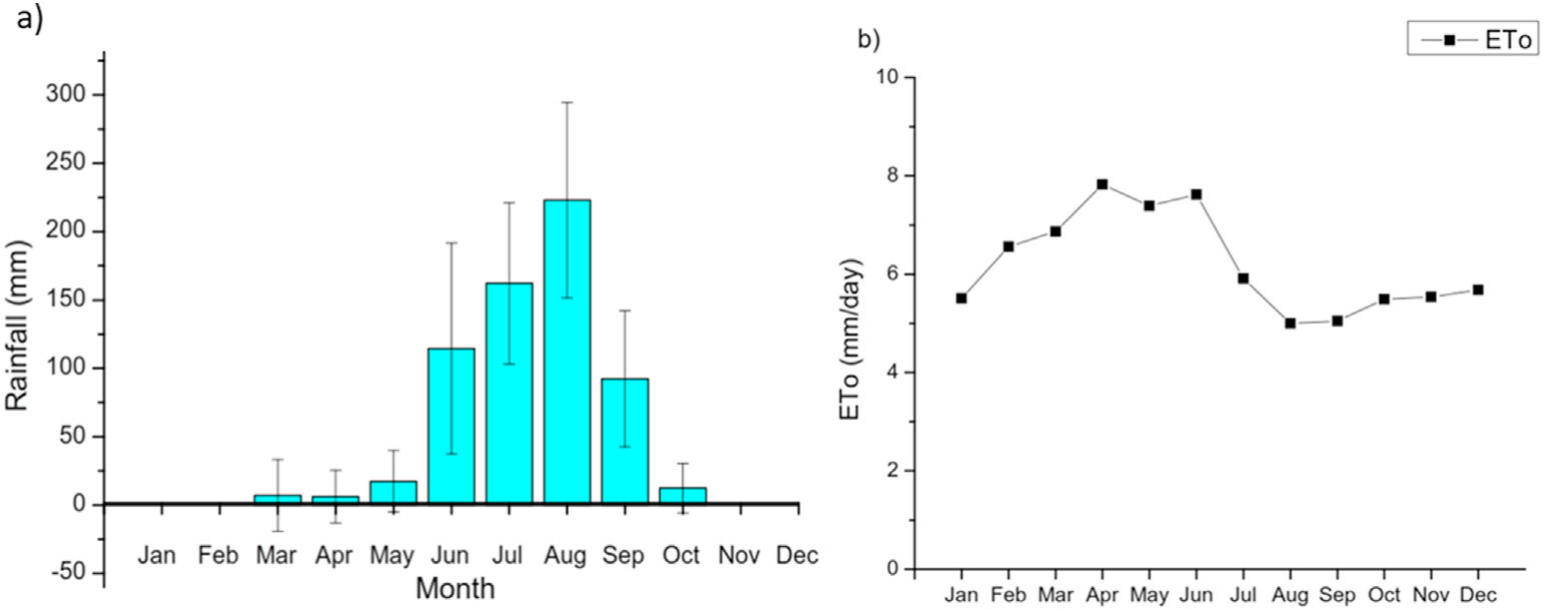
Mean monthly rainfall (a) and reference evapotranspiration (b) in the study site.

The effective rainfall was computed using the United States Department of Agriculture Soil Conservation Service (USDA-SCS) method built-in in the Food and Agriculture Organization (FAO) CROPWAT model. Similarly, reference evapotranspiration was calculated using the Penman-Monteith method (Eq. (2)) (Allen et al., 1998). The monthly values of rainfall and reference evapotranspiration are presented in Figure 4. The mean annual rainfall was 635 mm, out of which, more than 90% occurred in four months (i.e., June, July, August, and September). A monthly maximum rainfall of 223.1 mm was obtained in August. The reference evapotranspiration (ETo) was maximum during the months of May–June, amounting to 8 mm/day, with a minimum of 5 mm/day in August. The average ETo was found to be 6.3 mm/day. The monthly ETo ranged from 154 mm in August to 246 mm in May, with an average of 191 mm.

#### 2.4.2 Crop and soil data

The crop-related information, including planting dates, cropping pattern and yield were obtained via interviews with experts in HAM. The other inputs for CROPWAT model such as length of growth period, crop coefficients each growth stage, maximum rooting depth for each crop were obtained from Allen et al. (1998).

To identify soil types in the farm, soil samples were collected during the 2013/2014 season from 52 random points distributed over the whole farm. Analysis of soil parameters including soil texture, moisture contents at field capacity (FC) and wilting point (PWP), and bulk density were made at Mekelle University soil physics laboratory. The detailed crop and soil parameter values used by the model are presented in Table 3.

**Table 3:** Soil and crop characteristics used for CROPWAT model simulation.

Type	Parameter	Cotton	Mung-bean
Crop characteristics	Rooting depth (meter)		
	Minimum	1.00	0.60
	Maximum	1.70	1.00
	Length of growth period (days)		
	Initial stage	30	20
	Development stage	50	40
	Mid-stage	60	30
	Late-season stage	55	20
	Crop coefficients (Kc)		
	Kc-initial	0.35	0.4
	Kc-mid	1.15	1.05
	Kc-end	0.65	0.6
Soil characteristics	Soil texture	Clay	Clay
	Bulk density (g/cm^3^)	1.27	1.27
	Moisture content at field capacity (FC)	34%	34%
	Moisture content at wilting point (PWP)	25%	25%

### 2.5 The WF assessment

#### 2.5.1 WF based on CROPWAT model

The total consumptive water footprint (WF_T_) of a crop is the sum of the blue and green components of WF, as shown in Eq. (1) (Hoekstra et al., 2011).

(1) $$\text{WFT}={\text{ WF}}_{\text{B}}+{\text{WF}}_{\text{G}}\;\left({\text{m}}^{3}\text{/ton}\right)$$

where, WF_B_ is the blue WF, WF_G_ is the green WF.

The procedures followed to calculate the green and blue WFs are presented as follows.

First of all, the model computes the reference evapotranspiration based on F.A.O. Penman-Monteith method integrated within the CROPWAT model, as shown in Eq. (2) (Allen et al., 1998).

(2) $${\text{ET}}_{\text{O}}=\frac{0.408\Delta \left({\text{R}}_{\text{n}}-\text{G}\right)+\text{γ}\frac{900}{\text{T}+273}{\text{U}}_{2}\left({\text{e}}_{\text{s}}-{\text{e}}_{\text{a}}\right)}{\Delta +\text{γ}\left(1+0.34{\text{U}}_{2}\right)}$$

where: ET_o_ is reference evapotranspiration (mm day^−1^), R_n_ is the net radiation at the crop surface (MJ m^−2^ day^−1^), G is soil heat flux density (MJ m^−2^ day^−1^), T is mean daily air temperature at 2 m height (°C), u_2_ is wind speed at 2 m height (m s^−1^), e_s_ is the saturation vapor pressure (kPa), e_a_ is the actual vapor pressure (kPa), (e_s_ - e_a_) is the saturation vapor pressure deficit (kPa), Δ is the slope of vapor pressure curve (kPa °C^−1^), and γ is the psychometric constant (kPa °C^−1^).

The actual crop evapotranspiration (ET_a_) was computed as (Eq. (3)).

(3) $${\text{ET}}_{\text{a}}=\text{ Ks}{\text{ K}}_{\text{c }}{\text{ET}}_{\text{O}}$$

where, ETa is crop evapotranspiration (ET_a_) in mm/day, Kc is the crop coefficient (dimensionless) which varies depending on the type and growth stages of a given crop. The kc values for cotton and mung-bean crops were taken from Allen et al. (1998) as presented in Table 3. Ks, a dimensionless coefficient, has values ranging from 0 to 1, and refers to the condition of soil moisture and other factors such as salinity.

To estimate the green and blue components of crop evapotranspiration Eqs. (4) and (5) were employed (Hoekstra et al., 2011).

(4) $${\text{ET}}_{\text{G}}=\min\left({\text{ET}}_{\text{a}},{\text{ P}}_{eff}\right)\text{ and},$$

(5) $${\text{ET}}_{\text{B}}=\text{ max}\left(0,{\text{ ET}}_{\text{a}}-{\text{P}}_{eff}\right)$$

The effective rainfall (P_eff_) as the function of the monthly precipitation (P) (mm) was estimated using the Soil Conservation Service (SCS) method provided by the United States Department of Agriculture (USDA), as shown in Eqs. (6) and (7).

(6) $${\text{P}}_{\text{eff}}=\frac{\text{P∗}\left(125-0.2\text{∗}3\text{∗P}\right)}{125},\text{ for P}<=250/3\text{ mm, and}$$

(7) $${\text{P}}_{\text{eff}}=\frac{125}{3}+0.1\text{∗P},\text{ for P }<250/3\text{ mm}$$

The CWU (m^3^/ha) for each water-use type was calculated using Eqs. (8) and (9) (Aldaya et al., 2012).

(8) $${\text{CWU}}_{\text{G}}=10\text{∗}\sum_{\text{d}=1}^{\text{lgp}}{\text{ET}}_{\text{G}}$$

(9) $${\text{CWU}}_{\text{B}}=10\text{∗ }\sum_{\text{d}=1}^{\text{lgp}}{\text{ET}}_{\text{B}}$$

Where ET_G_ is the green water evapotranspiration and ET_B_ is the blue water evapotranspiration from day one (d = 1) until the specified length of the growth period (lgp). The factor, 10, is meant to convert water depths in millimeters into water volumes per land surface (m^3^/ha).

Finally, the green and the blue WF for each crop were determined using the method presented in Hoekstra et al. (2011) as shown in Eqs. (10) and (11). Both the blue and green WFs of a given crop were computed by dividing the crop water use (CWU) (m^3^/ha) of the crop by the yield (Y) (ton/ha).

(10) $${\text{WF}}_{\text{G}}=\frac{{\text{CWU}}_{\text{G}}}{{\text{Y}}_{\text{C}}}$$

(11) $${\text{WF}}_{\text{CB}}=\frac{{\text{CWU}}_{\text{B}}}{{\text{Y}}_{\text{C}}}$$

where, CWU_G_ and CWU_B_ are the green (rainfall) and blue (surface and groundwater) water uses by the crop. Y_C_ is the yield based on crop water requirements and actual evapotranspiration outputs from CROPWAT model. The linear relationship between crop yield and water stress proposed by Doorenbos and Kassam (1979) was used to estimate the yield.

#### 2.5.2 Blue WF based on field data

In computing the blue WF, the volume of irrigation water used to fulfill the deficit was considered as the blue water (Scarpare et al., 2016). The blue WF was calculated using Eq. (12).

(12) $${\text{WF}}_{\text{MB}}=\frac{10\left(\text{Ir}-\left(\text{DP}+\text{RO}\right)\right)}{{\text{Y}}_{\text{f}}}$$

where, WF_MB_ (m^3^/ton) is the blue WF estimated from measured irrigation data, Y_f_ is the yield of cotton and bean crops as obtained from field records, Ir is the irrigation water applied during the irrigation season (mm), DP is the deep percolation water leaving the root zone (mm), RO is surface runoff water. Since it is difficult to measure the losses from such large fields, an average irrigation efficiency of 70% was considered in the CP irrigation systems (Borsato et al., 2019) to account the lumped losses due to surface runoff and deep percolation.

### 2.6 Scenario development

To assess the effects of planting dates on WF, seven planting date scenarios (five hypothetical and two baseline scenarios) were assumed for cotton WF assessment. The five hypothetical scenarios (S1 to S5) correspond to dates from May 1 to June 1 at an interval of fifteen days (Table 4). The two baseline planting date scenarios were July 19 and July 24 corresponding to M-12 and M-6. For mung-bean, September 21 was taken as the baseline scenario for planting date. The planting date scenarios were developed with the assumption that changes in planting dates are manifested by the sole and combined effects of climatic variables on crop water requirements.

**Table 4:** Description of scenarios.

Scenario	Planting date	Description
Baseline	July 19 and July 24 for cotton September 21, for mung-bean	These planting dates were assumed to be the baseline scenarios, as these planting dates correspond to CP machines which were operational during the study year.
Scenario-1 (S-1)	May 1	The crops benefit from 98% of the annual rainfall but face with one of the highest monthly temperatures (40 °C) occurring in May and lower relative humidity at early stages of growth.
Scenario-2 (S-2)	May 15	The potential rainfall use is reduced to 96 % of annual rainfall, and faces the highset temperatures in May for a half of the month.
Scenario-3 (S-3)	June 1	Crop is faced with little reduction in rainfall and avoids the highest temperatures in May but the crop faces with the highest monthly wind speeds (3.2 m/s) in June affecting the early stages of growth.
Scenario-4 (S-4)	June 15	The crops could use 86% of the annual rainfall.
Scenario-5 (S-5)	July 1	The crops could use only 77% of the annual rainfall.

### 2.7 Statistical analysis

The blue WF estimates based on CROPWAT model and field data were compared using selected indices such as the normalized root mean square error (nRMSE), the normalized mean bias error (nMBE), and relative error (RE) as presented in Eqs. (13), (14), and (15). The use of normalized indices help to better evaluate the performance of a model (Karandish and Šimůnek, 2019)

(13) $$\text{nRMSE}=\frac{\sqrt{\frac{\sum_{i=1}^{n}{\left({\text{F}}_{\text{i}}-{\text{C}}_{\text{i}}\right)}^{2}}{\text{n}}}*100\%}{\bar{{\text{F}}_{\text{i}}}}$$

(14) $$\text{nMBE}=\frac{\frac{\left({\text{F}}_{\text{i}}-{\text{C}}_{\text{i}}\right)}{\text{n}}}{\bar{{\text{F}}_{\text{i}}}}*100\%$$

(15) $$\text{RE}=\frac{\left({\text{C}}_{\text{i}}-{\text{F}}_{\text{i}}\right)}{{\text{F}}_{\text{i}}}*100\%$$

where, F_i_ and C_i_ are the field-based and CROPWAT-based blue WFs, respectively, and n is the number of CP irrigated fields considered in this study.

In addition, the paired t-test method in the Statistical Package for Social Sciences (SPSS) version 20 was used to evaluate the statistical significance of the difference between the blue WF estimates based on CROPWAT model and field data.

## 3 Results

### 3.1 The WF of cotton and mung-bean

Based on the results from CROPWAT 8 (Table 5), the green and blue components of ET and WF (both in terms of yield and area) were calculated for the five cotton fields. The green water evapotranspiration (ET_G_) for cotton ranged from 213 mm for M-4C to 382 mm for M-12. Whereas, the blue water evapotranspiration (ET_B_) ranged from 514 mm for M-12 to 710 mm for M-4C. The average green, blue, and total WF of cotton were 967, 1778 and 2745 m^3^/ton, respectively. The total WF showed a small standard deviation as compared to its components, i.e., blue and green WF, which explains the lower variation in total WF among the five cotton fields. On the other hand, blue WF showed a high variation, with a standard deviation of 241.03 m^3^/ton.The higher difference in the blue and green WF, among cotton fields is more likely caused by the differences in climate variables (rainfall, temperature, wind speed, relative humidity, and sunshine hours) attributed to varying planting dates. The higher standard deviation of the blue WF hints the possibility for lowering the blue WF by introducing different management interventions.

**Table 5:** Statistical analysis of cotton crop WF for the five CP irrigated fields.

Parameter	Range	Minimum	Maximum	Mean	Std. Deviation
ET_G_ (mm)	169	213	382	319	68.16
ET_B_ (mm)	196	514	710	587	79.55
Green WF (m^3^/ton) (m^3^/ha)	511	645	1156	967	206.66
	1688	2127	3815	3192	682.16
Blue WF (m^3^/ton) (m^3^/ha)	594	1558	2152	1778	241.03
	1959	5142	7101	5867	795.14
Total WF (m^3^/ton) (m^3^/ha)	82	2714	2796	2745	34.36
	271	8957	9228	9059	113.34

The ET_G_ and ET_B_ for mung-bean were found to be 32.8 mm and 426.5 mm, respectively. The total WF of mung-bean was 6561m^3^/ton or 4593 m^3^/ha. The blue WF covers around 93 % of the total WF of mung-bean. The contribution of the green water was found to be very low for mung-bean because it was sown on 21^st^ September-commonly the end of the rainy season.

**Figure 5:**
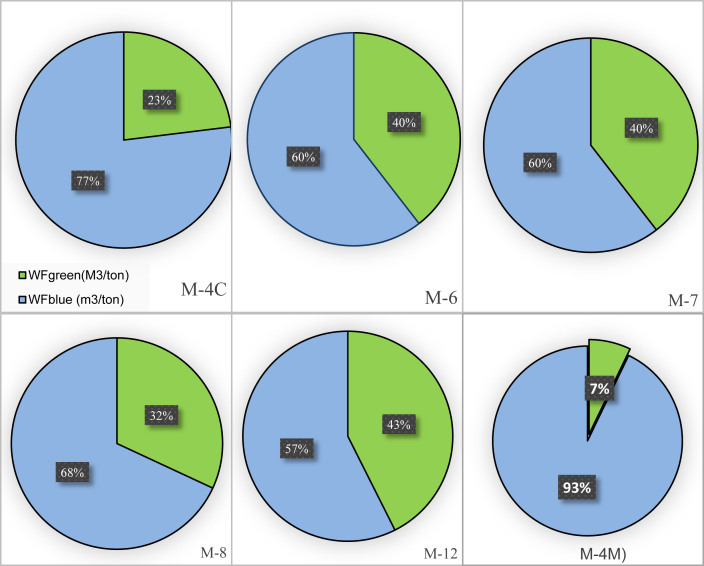
The blue and green WFs of Cotton (M-4C, M-6, M-7, M-8, and M-12) and mung-bean (M-4M) based on CROPWAT model.

Figure 5 shows the proportion of blue and green WFs for cotton and mung-bean crop production computed based on CROPWAT results for each area irrigated by the respective CP machine. The percentage of green WF of cotton ranged from a minimum of 23% (M-4C) to a maximum of 43 % (M-12). The cotton in M-12, sown on 19^th^ July 2017, resulted in a relatively higher percentage of green WF than M-4C, which was sown on 14^th^ August 2017. This could be due to the fact that M-12 might be benefiting from the higher rainfall amounts as it was sown earlier than others. The highest annual rainfall occurs in the months of June, July, August, and September, constituting about 18%, 25.5%, 35.1%, 14.5% of the average annual rainfall. For instance, a cotton field at M-4C, which was sown on 14^th^ August 2017 (M-4) would approximately loose around 60 % of the annual rainfall. Hence, the trend in higher blue WF percentages in all cotton fields is mainly due to the failure to harness the considerable rainfall amounts occurring in the rainy season.

### 3.2 Comparison of blue WF from CROPWAT against field-Data

The CROPWAT based blue WF (WF_CB_) estimates of cotton and mung-bean production were compared against the blue WF estimates derived from field-data based measurements (WF_FB_).

A paired t-test was conducted to compare the blue WF of cotton computed using the two approaches. The results showed that there is no significant difference (p = 0.456) in blue WF of cotton between WF_CB_ (mean = 1778, SD = 241) and WF_FB_ (mean = 1854, SD = 251) at p = 0.05. Moreover, the results also indicated lower values of error (nRMSE = 4.5 %; nMBE = 10.7 %) between WF_CB_ and WF_FB_ estimates of blue WF for cotton. Relative error/RE/values ranged from 0.8% to 17% for cotton, and 4.5% for mung-bean. These results suggested that CROPWAT model performed well in determining the blue WF of cotton. The good performance by the model could partly be attributed to the use of *in situ* input data such as soil texture, moisture content at field capacity and wilting point, bulk density, and rainfall.

**Figure 6:**
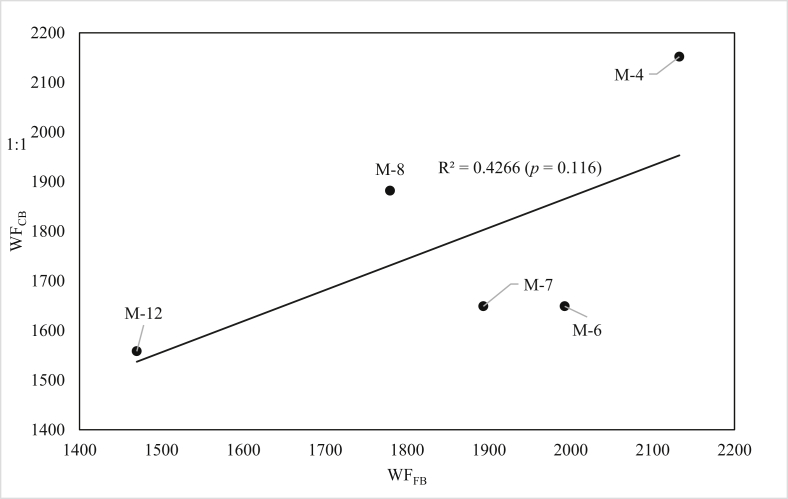
Correlation between blue WF estimates by WF_CB_ and WF_FB_ results.

Figure 6 indicates the correlation between WF_CB_ and WF_FB_ estimates for cotton. Visual inspection of the graph shows a non-consistent (over/under-estimation) of the blue WF by the WF_CB_ method. This is supported by a positive but non-significant (p = 0.116) correlation coefficient (r = 0.65) between CROPWAT based and field-based estimates. Overall, the results indicated that CROPWAT model could be used for estimating the blue WF for similar cases with special care on the input data to be used.

### 3.3 Effect of planting dates on WF

In this study, the effect of changes in planting dates on green, blue and total WF was investigated as a management strategy to save blue water. Traditionally, in the study site, the planting dates for cotton start in early to mid-June. The extent of variation in WF due to different planting date scenarios in comparison to the baseline scenarios are presented in Table 6.

**Table 6:** Variation (%) in blue, green and total WF of different planting date scenarios as compared to the baseline scenario.

Scenario	Green WF		Blue WF		Total WF	
	Cotton	Mung-bean	Cotton	Mung-bean	Cotton	Mung-bean
S-1 (May 1)	40.8	89.3	-56.2	-51.4	6.6	11.7
S-2 (May 15)	40.5	92.0	-71.4	-75.0	3.3	12.6
S-3 (June 1)	39.1	93.2	-74.9	-100.0	1.1	4.5
S-4 (June 15)	30.7	93.2	-45.0	-100.0	-0.1	5.1
S-5 (July 1)	13.1	92.3	-13.2	-97.8	-0.7	-4.6

#### 3.3.1 Effect of planting date on the WF of cotton

**Figure 7:**
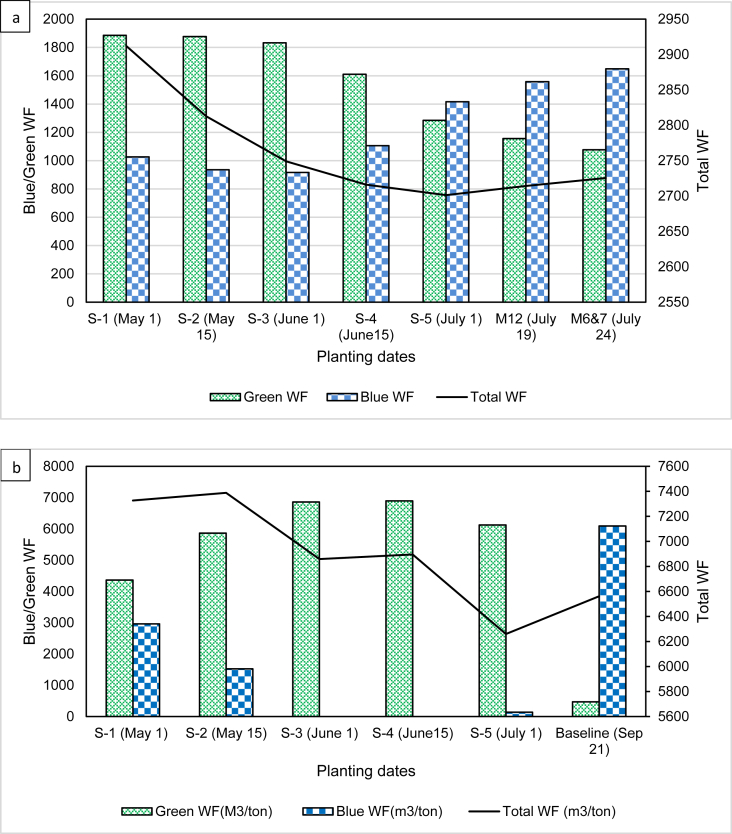
The effect of different hypothetical planting date scenarios on the total, blue and green WF of cotton (a) and mung-bean (b).

The results indicated that there is a descending trend in total WF (solid line) of cotton as the planting date shifts from May-1 (2912 m^3^/ton) to July 1 (2701 m^3^/ton) (Figure 7a). In comparison to the average of the two baseline scenarios, a very low variation in total WF, ranging from a 6.6 % increase for S-1 to a 0.7% decrease for S-5, was obtained (Table 6). Similarly, the green WF ranged from 1885.5 m^3^/ton (S-1) to 1284.5 m^3^/ton (S-5) exhibiting a decreasing trend with a corresponding variation of 40.8% and 13.1 %. However, the blue WF showed an increasing trend, ranging from 916 m^3^/ton to 1416 m^3^/ton. The reduction in blue water could be as high as 71.4–79.9% if planted in the days from May 15 (S-2) to June 1 (S-3) (Table 6). The rainy season mostly starts in June with the majority of it falling in the months of July and August. On the other hand, the temperature that occurs in May is among the highest in the area. Hence, cotton crops planted between the mid of May and early June are more likely to benefit from much of the green water while reducing the higher evapotranspiration rates due to the warmer temperature in May. Such changes in planting date resulted in lower blue water use which inturn imply the reduction in opportunity cost related to the use of blue water. Moreover, savings in blue WF might open a room for irrigating more land that would have not been used otherwise. In general, synchronizing the planting date changes along with the rainy season is believed to reduce the blue and total WF (Nyambo and Wakindiki, 2015).

#### 3.3.2 Effect of planting date on the WF of mung-bean

Figure 7b presents the green, blue and total WF of mung-bean in relation to changing planting dates. The total WF of mung-bean ranged from a minimum of 6260 m^3^/ton for S-5 to a maximum of 7388.6 m^3^/ton for S-2. The maximum variation in total WF in comparison to the baseline scenario was found to be 12.6% (Table 6). This variation corresponds to a planting date shift by almost two months earlier than the baseline scenario. The green WF was at its highest for S-3 (6858.5 m^3^/ton) and S-4 (m^3^/ton), implying that the crop water use (CWU) was solely covered by rainwater. The blue WF is highest when planted before May-1 (2962.8 m^3^/ton) but nil for S-3 and S-4 scenarios. This indicates that mung-bean planted in the month of June would benefit from the rainwater while avoiding the use of blue water. This could help save the whole blue water consumption by the crop and avoid the opportunity costs associated with the utilization of blue water.

## 4 Discussion

The WF assessment tool is appropriately used to find remedial measures for water resource management problems (Ercin et al., 2011). The capability of WF tool to compute blue and green WF enables to identify the amount and type of freshwater consumed for crop production. In this study, the WF of cotton and mung-bean was calculated using the CROPWAT model. Further analysis was conducted to compare the WF results against field-based data and to determine the effect of planting date on total, green and blue WFs.

### 4.1 Comparison with other studies

Results from the present case study indicated that the average total WF of cotton was 2745 m^3^/ton, in which the green WF and blue WF constitute 35% and 65 %, respectively. The WF of cotton estimates from previous studies are based on either local, national or global scale. As a result, the total WF results are widely varying. A study by Ahmed and Ribbe (2011) found a very high variation of total WF among irrigated cotton fields in five states of neighboring Sudan, which ranged from 5500 to 14000 m^3^/ton. Fu et al. (2019) also found a total consumptive WF of 6450 m^3^/ton. On a global scale, Mekonnen and Hoekstra (2011) estimated the total WF of cotton to be 3590 m^3^/ton, where the contribution of the green and blue is 2282 and 1308 m^3^/ton. On the contrary, Chu et al. (2017) and Zoidou et al. (2017) found a very lower total WF of 1493 m^3^/ton and 1491 m^3^/ton, respectively at a national scale. In comparison, the total WF estimates from this study tend to lay in between these two extremes. For mung-bean, the results from this research showed that the green, blue and total WF were 468 m^3^/ton, 6093 m^3^/ton, and 6561 m^3^/ton, respectively. Gheewala et al. (2014) estimated the WF of mung-bean production in Thailand to be in the range of 1549–6445 m^3^/ton. The maximum from these results are close to the WF results from the present study. Overall, the variation between this study and other studies could be due to many factors such as spatial resolution of datasets used, season (irrigated or rain-fed), types of simulation models employed, crop species and duration of growth periods, planting dates, soil parameters and climatic variabilities.

### 4.2 The use of crop models in WF assessment

To reduce the time and costs required to conduct field experiments, researchers tend to use mathematical/conceptual models to compute the WF of crop production (Herath et al., 2014). Many studies have reported the successful use of such models for WF assessments at global, national and local scales (Ahmed and Ribbe, 2011; Aldaya et al., 2010; Chapagain et al., 2006; Jin et al., 2016; Nana et al., 2014; Tsakmakis et al., 2018) or at field level (Gobin et al., 2017; Herath et al., 2014). However, model-based estimates WF of crop production are often criticized for associated uncertainties.

In the present study, an attempt was made to assess the performance of CROPWAT in determining the blue WF of cotton production. The paired t-test showed no significant difference between the model and field-based estimates. Moreover, the model performed well with nMRSE, nMBE values of 4.5 % and nMBE = 10.7 % respectively, and the relative error ranged from 0.8 to 17% was found. Overall, CROPWAT model performed well in estimating the blue WF. However, the relatively lower Pearson correlation coefficient (0.65) indicated the limitation in fully relying on the model results. Tsakmakis et al. (2018) successfully employed both CROPWAT and AquaCrop models to assess the effect of different management strategies on WF crop production. Other researchers have also employed other models to estimate either the total WF or components of WF for a variety of crops in different parts of the world. For instance, Karandish and Šimůnek (2019) compared two model-simulated results (HYDRUS 2D/3D) and SALTMED models) against field-based gray WF from maize production and found closely related values. The use of WF assessment results from models that use climate data for small irrigation areas minimizes the time and energy. However, such models need to be validated using field data before they are used for WF assessment, especially in small scale assessments.

### 4.3 Implications of reducing blue water consumption

In this study, we simulated the effect of planting date, as a management strategy, on the WF of crop production with specific emphasis on blue WF reduction. The effect of planting date on the total WF varied among selected planting date scenarios. The reduction in blue WF due to a shift in planting date could be as high as 79.9% for cotton and up to 100 for mung-bean. Previous studies have also reported a significant decrease in blue WF by changing planting dates. For instance, a study by De Miguel et al. (2015) reported a 50% reduction in the blue WF of wheat due to a 30 days earlier planting dates. Zhuo et al. (2014), found up to a 40% reduction in blue WF of maize production due to late planting date. Tuninetti et al. (2015) have also indicated a reduced total WF due to a delay in planting date of winter wheat. These show that a substantial amount of blue WF could be saved by making the planting dates close to early June. Moreover, there is a scarce availability of irrigation water in the area.

Reduction in the total and blue water consumption as a water scarcity alleviation mechanism had been implemented using different approaches in previous studies. Nouri et al. (2019) tested the impact of soil mulching and drip irrigation, and their combined effect which led to about a 5% reduction in blue water consumption. Another study by Chukalla et al. (2015), evaluated the effect of different irrigation techniques, irrigation strategies, and mulching and managed to obtain up to 44 % reductions in blue WF. Similarly, Tsakmakis et al. (2018) obtained a 5–12 % reduction in WF of cotton by applying different irrigation technologies and strategies.

Any mechanism to minimize the use of irrigation water, and primarily the blue water, would be advantageous in terms of water management and increasing overall production. Blue water plays a major role in crop production in rainfall scarce regions of the world. Recently, in Ethiopia, many water development projects are in place in order to supply water for irrigation in particular and increase food production in general. However, unlike the progress in blue water development projects, water management in countries like Ethiopia is very poor. On the other hand, blue water is associated with more opportunity costs than green water. Thus, studies conducted to improve blue water consumption have a considerable implication on the use and management of the scarce resource and overall economy of a country.

### 4.4 Limitations of the study

In this study, the CROPWAT model accompanied by field-based data were employed to assess the WF and the effect of planting date on WF of crop production. WF assessment based on field data is very important in reducing the uncertainties in WF assessment from model simulations. Hence, an attempt was made to validate the model based on field data, mainly irrigation depth and yield data. However, our field data was only limited to the computation of blue WF. Moreover, to get a more reliable results, it should be supported by green WF obtained from field-based inputs such as actual evapotranspiration (measured using Lysimeters) and other water balance components. Another limitation of this study is that the WF assessment of two crops in a single year made it difficult to support the results with strong statistical analysis. Hence, future research should consider WF assessment involving more cases, both temporally and spatially, for better results.

## 5 Conclusions

In this case study, we assessed the WF of cotton and mung-bean crops in CP irrigated farms in northern Ethiopia using CROPWAT based results. We also compared the CROPWAT based blue WF against field-data based estimates. The average total, green and blue WF of cotton from five CP fields were 967, 1778 and 2745 m^3^/ton, respectively. For mung-bean, the total, green and blue WF were found to be 6561m^3^/ton, 468 m^3^/ton, and 6093 m^3^/ton, respectively. The comparison in blue WF of cotton from CROPWAT and field-data showed a good agreement (nRMSE = 4.5 %, nMBE = 10.7 % and RE ranging from 0.8 to 17%). A paired t-test analysis also supported the non-significant difference between the means of the two estimates. From the results, we can conclude that CROPWAT has performed well in estimating the blue WF of cotton and mung-bean crop production. However, low correlation coefficient (r = 0.65) that might suggest the limitation of the CROPWAT model in accurately estimating the blue WF.

In addition, an attempt was made to investigate the effect of planting date on the total, blue and green WFs based on climate data. The variation in total WF among the baseline and different planting date scenarios was 0.7%–6.6 % for cotton and up to 12% for mung-bean. However, the variation in blue WF was high with a range from 13.2-79.9% for cotton and up to 100 % for mung-bean. These variations indicate the room for reducing the blue WF of both crops as a result of changes in planting date. Hence, in the area, shifting planting dates would have a considerable effect in saving the blue water use which in turn is an opportunity for crop intensification and extensification.

Assessment of WF of crop production has a significant role in managing the scarce freshwater resources. In our study, we have assessed and characterized the existing water use of cotton and mung-bean from different sources of water using two different approaches. Further options to minimize the scarce blue water resource were also discussed. Future studies should focus on the use of various irrigation and agronomic management strategies to reduce freshwater use by crops.

## Declarations

### Author contribution statement

Filmon Tquabo Gebremariam: Performed the experiments; Analyzed and interpreted the data; Wrote the paper.

Solomon Habtu: Analyzed and interpreted the data; Wrote the paper.

Eyasu Yazew: Conceived and designed the experiments; Wrote the paper.

Berhane Teklu: Analyzed and interpreted the data; Contributed reagents, materials, analysis tools or data; Wrote the paper.

### Funding statement

This work was supported by Hiwot Agricultural Mechanization (HAM) and 10.13039/501100009402Mekelle University.

### Data availability statement

Data will be made available on request.

### Declaration of interests statement

The authors declare no conflict of interest.

### Additional information

No additional information is available for this paper.
